# Psychosocial Stress and Age Influence Depression and Anxiety-Related Behavior, Drive Tumor Inflammatory Cytokines and Accelerate Prostate Cancer Growth in Mice

**DOI:** 10.3389/fonc.2021.703848

**Published:** 2021-09-16

**Authors:** Denise L. Bellinger, Melissa S. Dulcich, Christine Molinaro, Peter Gifford, Dianne Lorton, Daila S. Gridley, Richard E. Hartman

**Affiliations:** ^1^Department of Pathology & Human Anatomy, School of Medicine, Loma Linda University, Loma Linda, CA, United States; ^2^Department of Psychology, School of Behavioral Health, Loma Linda University, Loma Linda, CA, United States; ^3^Department of Psychology, Kent State University and the Kent Summa Initiative for Clinical and Translational Research, Summa Health System, Akron, OH, United States; ^4^Departments of Radiation Medicine and Biochemistry and Microbiology, School of Medicine, Loma Linda University, Loma Linda, CA, United States

**Keywords:** psychosocial stress, aging, tumor immunity, IL-9/IL-17 balance, anxiety/depression-related behavior

## Abstract

Prostate cancer (PCa) prevalence is higher in older men and poorer coping with psychosocial stressors effect prognosis. Yet, interactions between age, stress and PCa progression are underexplored. Therefore, we characterized the effects of age and isolation combined with restraint (2 h/day) for 14 days post-tumor inoculation on behavior, tumor growth and host defense in the immunocompetent, orthotopic RM-9 murine PCa model. All mice were tumor inoculated. Isolation/restraint increased sympathetic and hypothalamic-pituitary-adrenal cortical activation, based on elevated serum 3-methoxy-4-hydroxyphenylglycol/norepinephrine ratios and corticosterone levels, respectively. Elevated zero maze testing revealed age-related differences in naïve C57Bl/6 mice, and increased anxiety-like behavior in tumor-bearing mice. In open field testing, old stressed mice were less active throughout the 30-min test than young non-stressed and stressed, and old non-stressed mice, suggesting greater anxiety in old stressed mice. Old (18 month) mice demonstrated more depression-like behavior than young mice with tail suspension testing, without effects of isolation/restraint stress. Old mice developed larger tumors, despite similar tumor expression of tumor vascular endothelial growth factor or transforming growth factor-beta1 across age. Tumor chemokine/cytokine expression, commonly prognostic for poorer outcomes, were uniquely age- and stress-dependent, underscoring the need for PCa research in old animals. Macrophages predominated in RM-9 tumors. Macrophages, and CD4^+^ and CD4^+^FoxP3^+^ T-cell tumor infiltration were greater in young mice than in old mice. Stress increased macrophage infiltration in old mice. Conversely, stress reduced intratumoral CD4^+^ and CD4^+^FoxP3^+^ T-cell numbers in young mice. CD8^+^ T-cell infiltration was similar across treatment groups. Our findings support that age- and psychological stress interacts to affect PCa outcomes by interfering with neural-immune mechanisms and affecting behavioral responses.

**Graphical Abstract:**
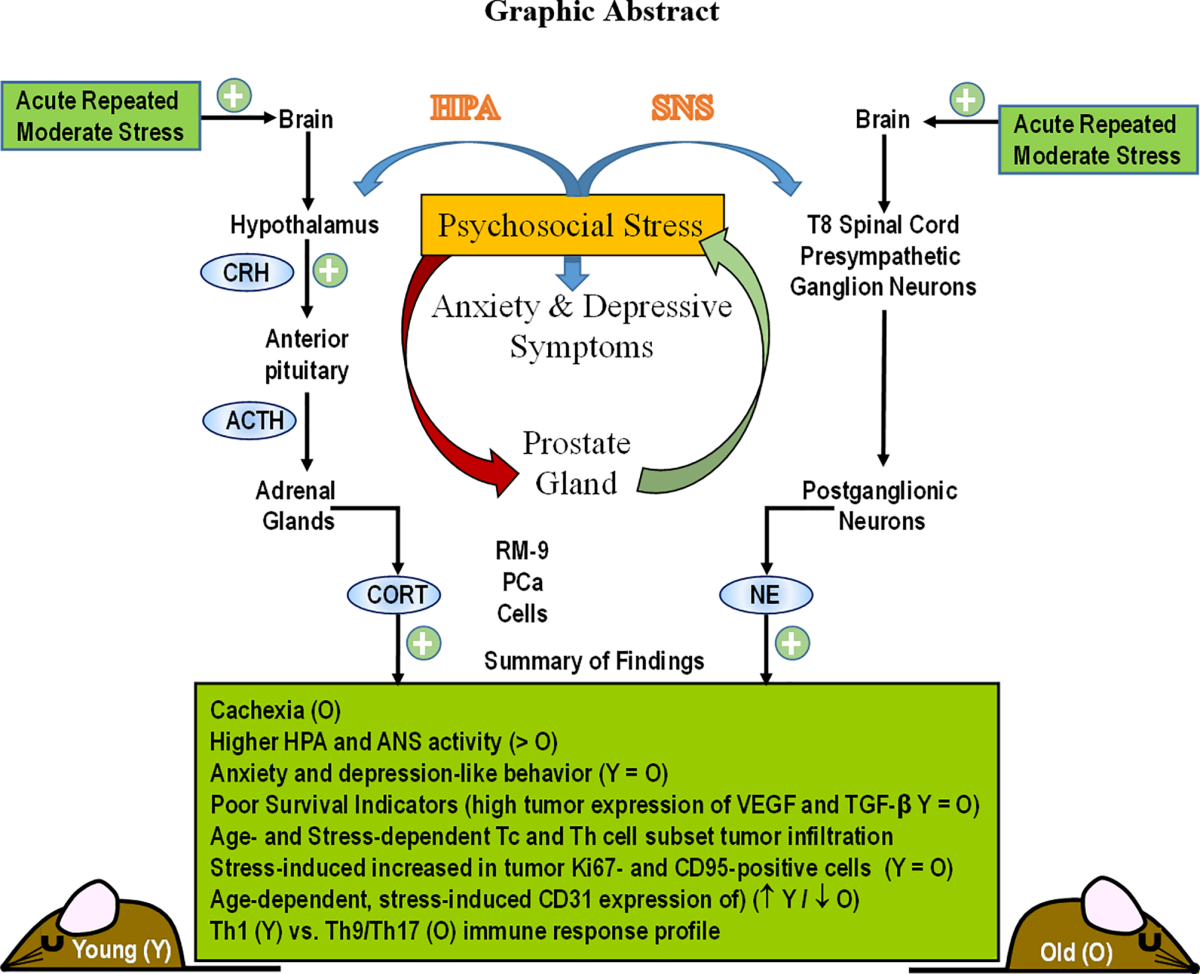


## Introduction

Prostate cancer (PCa) is the most prevalent cancer, and third most common cause of cancer-related death in men (1). Psychological stress and depression, which can alter the expression of cancer-linked genes in the prostate, is prevalent in patients with PCa (2). PCa incidence is directly linked to patient age, and perceived stress can increase with increasing age (3). Studies investigating the effects of age- and psychosocial stress-related changes on the microenvironment of PCa is limited, despite PCa being the most prevalent cancer in men. In this population there is a five-year relative survival rate for all stages of PCa. The 5-year survival rate for men with metastatic PCa is only 30% (4). Identifying mechanisms in which psychosocial stress affects the pathophysiology and disease outcomes of PCa in animal models may lead to improved patient care.

Anxiety and depression are major challenges for PCa survivors, particularly in the first 5 to 10 years post-cancer diagnosis (5–8). However, men rarely seek mental health care (5), despite that depression negatively impacts survival of men with metastatic PCa (6). Acute repetitive or chronic stress, anxiety and depression are relatively high in men with PCa (8), and may be predictive of cancer progression and/or mortality (9). Murine models of stress have significantly advanced our knowledge of mechanisms responsible for stress-induced changes in inflammation and immunity in other types of cancer, but research in this area for PCa is comparatively limited (2, 6–10).

Mood disorders in cancer survivors are proposed to evolve from combinations of tumor pathophysiology, cancer interventions, and stress (10). The impact of each of these is difficult to dissect out in the clinical setting. Animal research can control for confounding variables difficult to control for in clinical settings and may be used to disentangle mechanistic interactions between neural, immune and endocrine processes. Among these factors are chronic inflammation and anti-tumor immunity that are reported to correlate with fatigue and persistent negative affect (11). Mood, comorbidities and inflammation exist before cancer diagnosis and treatments (12, 13).

Hypothalamic-pituitary-adrenal (HPA), vagal nerve, and sympathetic nervous system (SNS) activity and their response to cancer- and/or treatment-related challenges are altered in cancer patients (14–19). Age-related changes in autonomic innervation of the prostate gland and changes in nerve activity also can influence PCa development and progression (15, 19). Autonomic and HPA pathways exert potent anti-inflammatory actions and influence behavior (20, 21). Mechanistic interactions between these factors remain unresolved.

Rodent models are the mainstay research tools to systematically identify the etiology of behaviors comorbid with cancer. Psychosocial stressors promote prostate carcinogenesis in mice that is sympathetically-mediated *via* regulation of anti-apoptotic signal pathways (22). For example, restraint stress altered the expression of cancer-related genes in the prostate (2). Herrara-Corvarrubias et al. (23) reported that an immune stressor during puberty promotes precancerous lesions in adult rats. Decker et al. (24) found that the SNS reactivated quiescent PCa cancer cells and promoted their metastases to the bone marrow. This research supports the idea that psychosocial interactions can significantly influence prostate physiology and PCa progression, consistent with breast cancer research that supports stress as an important moderator of tumor progression (25–27).

Preclinical research has targeted neural-immune-mediated mechanisms in tumor biology. Altered neural functions that manifest as depressive and anxiety-like behaviors are present in many rodent models of solid tumors (28–30). Given the rising and aging population of cancer survivors, it is important to understand the behavioral consequences of a cancer diagnosis and progression that contributes to disease pathophysiology.

One hallmark of the aging prostate is tissue remodeling and greater inflammatory cell infiltration that contribute to the age-related pathology observed in the prostate (31–33). Anti-tumor immunity can markedly differ in prostate tumor models using young or old mice. During aging, both molecular and structural changes develop to disrupt matrix components, and promote a proinflammatory microenvironment. These changes include stromal proliferation, robust T cell and macrophage infiltration and up-regulated proinflammatory cytokines and growth factors that are contributory to benign hyperplasia, prostatitis, and PCa (34). Remodeling of the extracellular matrix in the aged prostate microenvironment is also linked with greater PCa growth and invasion. Compared with young mice, prostate tumor cells orthotopically inoculated into the prostate, grow at an accelerated rate in old mice (31–35). Taken together, these findings demonstrate an aged prostatic microenvironment whereby resident immune cells, particularly macrophages and their polarization, adopt a protumorigenic phenotype that collaborates with the extracellular matrix to advance PCa in aging mice, and by extension aging men. Understanding the regulation of key mediators of PCa progression in the tumor microenvironment of the aged prostate is necessary to improve treatment of elderly men with PCa.

The aim of this study was to use an age-appropriate, syngeneic, immunocompetent, orthotopic animal model of PCa to evaluate the effects of age and chronic psychological stress that induces anxiety- and depressive-like behaviors on cancer progression and anti-tumor immunity. Using the RM9 prostate cancer cell line, a murine prostate reconstitution (MPR3) model was established young and aging male C57BL/6 mice for this purpose. In this paper, we report significant effects of both age and chronic stress on (1) depression-like behaviors, (2) stress pathway activation, (3) PCa progression based on local measures of proliferation, cell death, vascularization, and immune cell infiltration into RM-9 tumors cell. Collectively, our findings indicate that both age and psychosocial factors can interact to affect anti-tumor immunity and PCa outcome.

## Materials and Methods

### Animals

**Figure 1:**
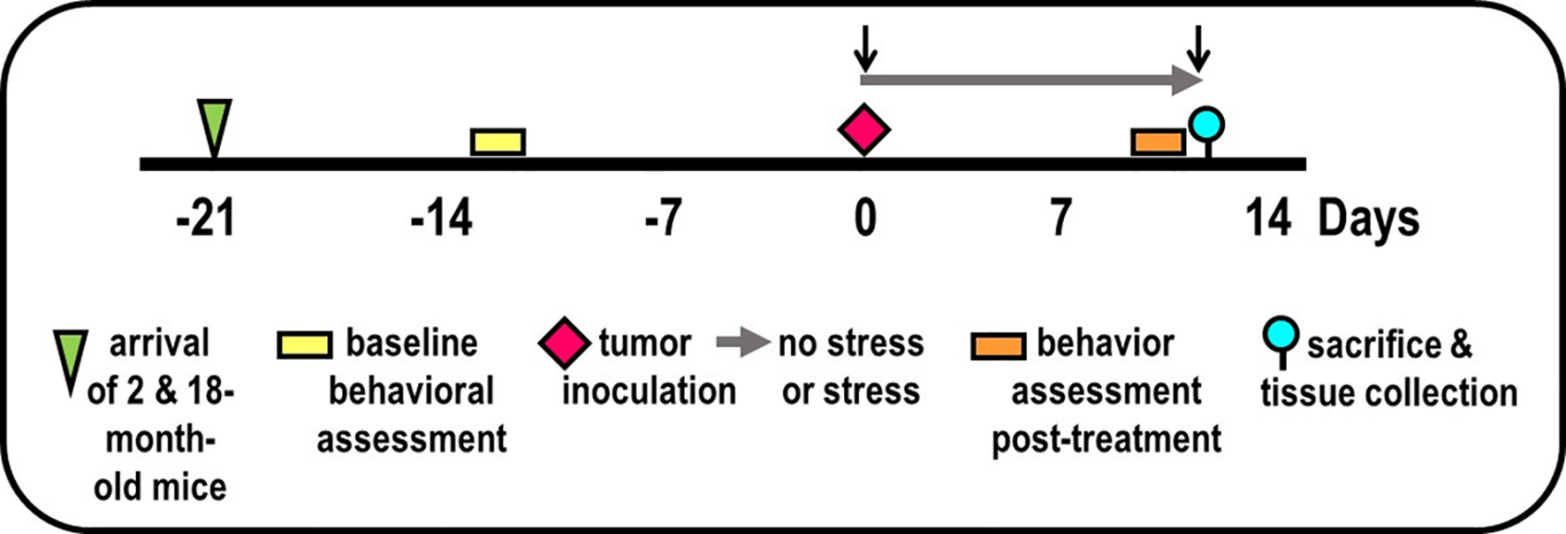
Experimental Design. Experimental timeline of events is illustrated, including mice arrival (triangle), accommodation to vivarium conditions, pre- and post-treatment behavioral testing (yellow/orange bars, respectively), tumor inoculation (diamond), collection of blood by retroorbital bleeding (↓), and tissue collection for endpoint assessments (circle).

Two- and 18-month-old male C57BL/6 mice (young and old, respectively) were purchased from the NIA colony (Charles Rivers Laboratories, Wilmington, MA). Upon arrival, mice were housed 4-5 per cage in the vivarium at Loma Linda University; Mice shipped in the same containers were housed together to minimize fighting, and were acclimated to vivarium conditions for 1 week (temperature, 22 ± 1°C; humidity, ~50%; 12-h light/dark cycling, environmental enrichment, and food and water provided *ad libitum*). Mice were then acclimated to handling for one week to minimize distress during the study, and then inoculated orthotopically with syngeneic PCa cells (**Figure 1**). Mice were observed for general health throughout surgery and post-surgery recovery for tumor inoculation. Feeding, drinking, and grooming behaviors were monitored and recorded. Animal procedures were approved by the Institutional Animal Care and Use Committee, in compliance with the National Institutes of Health (NIH) Guide for the Care and Use of Laboratory Animals.

### RM-9 Cells

The mouse prostate reconstitution or MPR^3^ model system using RM-9 prostate tumor cells was chosen to closely mimic complex, morphological, immunological, and molecular changes that underlie PCa (36–38). RM-9 tumor cells have similar mutations or aberrant activities of ras, myc, and p53 as in human PCa cells (36–38), and a low MHC class I profile as in many human PCa cell lines (36–38). The RM-9 PCa cell line is derived from a ras^+^ myc transformed/wild-type p53 primary prostate tumor induced in the Zipras/myc-9-infected C57BL/6 murine prostate reconstitution (MPR^3^) model (36–38). RM-9 cells were generously provided by Dr. Timothy C. Thompson at the Baylor College of Medicine in Houston, TX.

RM-9 cells were grown in Dulbecco’s modified Eagle’s medium (DMEM) with high glucose and L-glutamine (GIBCO, Grand Island NY) and supplemented with 10% fetal bovine serum (Omega Scientific Inc., Tarzana, CA), 10 mM HEPES buffer (Hyclone Laboratories, Inc., Logan, UT), 50 international units (IU)/ml penicillin and 50 µg/ml streptomycin (Mediatech Inc., Manassas, VA) at 37°C in a humidified atmosphere containing 5% CO_2_. RM-9 cells were passaged by trypsinization with 0.05% trypsin/0.53 mM EDTA in HBSS without sodium bicarbonate, calcium or magnesium (Mediatech Inc., Manassas, VA). RM-9 cells were counted, viability assessed using the trypan blue exclusion method (Sigma-Aldrich, St. Louis, MO) and then resuspended in medium. RM-9 cells were frozen at passage 14, thawed out and cultured prior to each experiment.

### Tumor Induction

RM-9 cells were trypsinized, washed 1X with 10 ml DMEM, resuspended in medium and counted. The viability was 90-94%. One ml of cells, adjusted to a concentration of 5 x 10^5^ cells/ml, were centrifuged at 200g for 8 min at 4°C. Supernatants were discarded, and the cells resuspended in 1 ml sterile saline.

After vivarium accommodation and baseline behavioral evaluation (**Figure 1**), mice were anesthetized with sodium pentobarbital. A low transverse abdominal incision was made, and the dorsolateral prostate was exposed. A 10-μl suspension of 5,000 RM-9 cells was injected into the dorsolateral prostate to induce an *in situ* primary prostate adenocarcinoma in immune-competent mice (37). The incision was closed with wound clips. The viability of the RM-9 cells used for inoculation was 83-89% post-tumor inoculation. To control for surgery effects, (i.e., tissue repair, anesthesia), additional mice (*n*=8) that received no treatment or that were inoculated with the vehicle minus RM-9 cells were included in the study to control for surgical effects on behaviors assessed in this study.

### Affective-Like Behavior

Two standardized tests of anxiety-like behavior (open field test (OFT)) and elevated zero maze (EZM)) and one test of depressive-like behavior (tail suspension test (TST)) (39) were administered in that order over 3 consecutive days between 900-1100 h Pacific Standard Time. This affective-like behavior battery was administered D14 before tumor inoculation and prior to the study endpoint (see **Figure 1**). Two researchers, who were blinded to the treatment groups, scored all behavior videos. Prior to restraint-stressing, mice were tested for baseline stress activity in the EZM. Post restraint-stressing, anxiety and learned helplessness were tested with the EZM, OFT, and the TST.

#### Anxiety-Related Behavior

The EZM consists of a 10-cm wide circular plastic track (100-cm outer diameter and elevated off the floor) with 35-cm tall walls enclosing 2 opposing quadrants. The room lights were dimmed, and halogen lights directly illuminated the open spaces of the maze. Animals were initially placed in the center of one of the open quadrants and their activity was monitored for 5 min. Time spent within the enclosed quadrants was calculated.

#### General Activity Levels/Movement Patterns

The OFT was used to assess anxiety-like behaviors and locomotion. Each animal was placed in a 49x36-cm^2^ opaque open-topped plastic bin for 30 min. The movements of each animal were recorded by an overhead camera and analyzed by a computerized tracking system (Noldus Ethovision, Leesburg, VA). A loose layer of bedding was added, and the arena was cleaned with 10% bleach between mice. The distance the animal moved, percent time spent moving, time spent in the perimeter and center of the test area were measured.

#### Learned Helplessness/Depression

The standardized TST assesses depression-like behaviors and learned helplessness (40). The animal is placed in an inescapable, uncomfortable situation, and immobility (lack of struggling) is measured. Mice were suspended for 6 min by the tail with adhesive tape attached approximately 1 cm from the tip of the tail. The other end of the tape was wrapped around a hook embedded in the center of the ceiling of a wooden box measuring 19L x 21W x 40H cm. When suspended, the animal’s nose was approximately 20 cm from the floor. The box was partitioned and enclosed on all sides except one (for viewing). Room lighting and sound were kept to a minimum. A partition visually isolated each mouse.

While the animal struggled to escape its position, two researchers blinded to treatment group individually rated the mouse on immobility and agitation. The percent time immobile was calculated for the final 4 min. Immobility was defined as a complete lack of voluntary movement by the mouse. An animal was also rated immobile if it was curled up, appearing to rest while holding its front paws to its back paws, but was not struggling or moving.

### Experimental Design

Two replicate experiments were performed, each with 25 mice per age per experiment (100 mice total), i.e., the maximum number per purchase by NIA. Mice were assigned to groups based on (1) obtaining equivalent mean and variance for baseline EZM performance (→) between treatment groups (to reduce the possibility of pre-existing differences in affective behaviors), and (2) maintaining existing housing for group-housed mice. Maintaining housing conditions for group-housed mice alleviated the effects that housing rearrangement would have on aggressive behavior and fighting between cage mates. Baseline behavioral testing (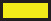) using the EZM was performed 1-week after arrival (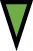) of mice to the vivarium (**Figure 1**). On D0, mice were anesthetized, blood was collected by retro-orbital bleeding (↓), then tumor cells were orthotopically implanted into the prostate (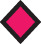) in all mice. After surgery, mice in the non-stressed group were returned to their home cage (i.e., grouped), whereas mice in the stressed group were place individually in a novel cage (**→**). Mice were weighed every 2 days. Young and old tumor-bearing mice were group-housed (non-stressed) and isolated/restraint-stressed (stressed). On D1, individually housed mice were restrained by placing the animal in well-ventilated, capped PVC tubes (2.54 cm in diameter for young and 3.175 cm in for old) for 2 h/day for 13 days. For each day of restraint stress, the restraint was randomized both in time of day and order of mice. Before restraint stress on D13, EZM testing (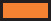) was performed on all mice. On D14 (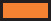), all mice were evaluated with the OFT, followed by the TST. At the time of sacrifice (D15), mice were weighed and then euthanized by an overdose of Nembutal (50 mg/kg, i.p.), bled retro-orbitally within 5 min after injection, and targeted tissues collected dissected (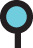).

### Tissue Collection and Tumor-Related Assessments

After cardiac puncture, blood was collected (800-1100 h PST) in heparin-coated syringes. Serum glucocorticoid and catecholamines were quantified using an enzyme-linked immunosorbent assay (ELISA) or high-performance liquid chromatography with coulometric detection (HPLC-CD). Spleens and tumors were dissected, weighed and frozen on dry ice. The brain and visceral organs (lung, liver, adrenal and pituitary glands) were grossly examined for age-related tumors or overt pathology. No age-related pathologies were observed in 18 month-old C57Bl/6 mice, and as previously reported for young mice (36–38), RM-9 tumors were non-metastatic in old mice.

Body, spleen, and tumor weights, circulating stress hormones, relevant organ weights, and tumor cytokine expression were end-point measures. The *n*’s were 10 mice per non-stressed or 15 mice per stressed treatment groups per replicate study. Primary tumor and organs with metastatic potential – the pelvic and retroperitoneal lymph nodes that drain the tumor site, femur bone marrow and lungs, were dissected and weighed. Tissues were fixed in 10% buffered formalin, paraffin embedded, cut on a rotary microtome at 5 µm, and stained with hematoxylin and eosin (H&E) for light microscopy. A piece of the primary tumor was immunohistochemically-stained for specific immune cell subsets including T-helper, T-regulatory, T-cytotoxic cells, and F480, M1 and M2 macrophages.

### Serum Stress Hormones

#### Corticosterone

To determine treatment group differences in corticosterone, blood from retro-orbital sinus bleeding was centrifuged at 2,000*g* for 10 min at 4°C, and serum was aliquoted and stored at -80°C. After thawing and diluting samples 1:25 with assay diluent, duplicate serum samples were assayed for corticosterone using an AssayMax ELISA kit following manufacturer’s instructions (AssayPro, St. Charles, MO; minimal detection level: 40 pg/ml; intra-assay and inter-assay coefficients of variation: 5.0 and 7.0%, respectively). Samples with a coefficient variance greater than 15% were repeated. Absorbance was read on a microplate reader at wavelengths of 450 and 570 nm immediately after adding the stopping solution. A wavelength correction was made by subtracting readings at 570 nm from those at 450 nm to correct for optical imperfections. Corticosterone concentrations were determined from standard curves generated from serially diluted standards run in duplicate on each plate.

#### Catecholamines

Serum catecholamine concentrations were determined after alumina extraction by HPLC-CD using a CouleChem HPLC System (ESA, Chelmsford, MA). The peak heights and area under the curves were analyzed using EZChrom Elite Software (Scientific Software Inc., Pleasanton, CA). Known standards for norepinephrine, dopamine, epinephrine, and the norepinephrine catabolite, 3-methoxy-4-hydroxyphenylglycol (MHPG) were used to determine sample levels and were corrected for recovery using 3, 4-dihydroxybenzylamine as the internal standard. The ratio of norepinephrine-to-MHPG concentration served as an index of norepinephrine turnover and SNS activity.

### Tumor Growth Factor, Chemokine and Cytokine Expression

We evaluated tumor expression of growth factors, chemokines and cytokines known to be prognostic for poorer prostate cancer outcomes, as well as screening for novel immune markers that may potentially influence tumor growth. Frozen prostate tissue samples were homogenized using a PowerGen 125 tissue homogenizer (Fisher Scientific, Pittsburg, PA). Ten μl of 10 mM Tris lysis buffer (pH 7.5) containing protease inhibitors (One Complete Mini tablet; Roche Mannheim, Germany) per 10 ml of buffer per mg tissue were added to each tissue sample. Samples were homogenized on ice. Homogenates were centrifuged in 1.5-ml Eppendorf tubes (4°C, 12,000*g* for 10 min). The supernatants were aliquoted into prelabeled 0.5-ml Eppendorf tubes and frozen at -80°C.

For TGF-β1, a 25-µl aliquot of prostate tissue homogenate of each sample was diluted with 75 µl of the Tris Lysis Buffer. Ten µl of 0.1 M HCl was added, per the manufacturer’s recommendation (R&D Systems, Minneapolis, MN). The samples were briefly vortexed, and after 10 min, neutralized with 13 µl of a 1.2 M NaOH/0.5M HEPES solution for a final dilution factor of 4.92. The samples were assayed in 96-well plates using Quantikine TGF-β1 ELISA kits (R&D Systems). The optical density of each well was read within 30 min of adding the stop solution using a microplate reader set at 450 and 540 nm. Samples were run in duplicate. Samples with a coefficient variance greater than 15% were repeated. The average TGF-β1 concentrations from the duplicate sample readings were determined from the values of standards present in each 96-well plate. The lower limit of detection for TGF-β1 was 4.61 pg/ml (R&D Systems).

For tumor cytokine expression, multiplexed immunoassay kits were employed. Prostate tumors were homogenized in protein extraction buffer [phosphate-buffered saline (PBS)], 0.05% Triton-X, Halt™ Protease Inhibitor Cocktail (Thermo Fisher Scientific, Waltham, MA) using acid-washed 1.4-mm zirconium beads and a benchtop BeadBug™ tissue homogenizer (Benchmark Scientific, Sayreville, NJ). Homogenates were sonicated for 1 min in a sonication bath (Branson M1800, Branson Ultrasonics, Danbury, CT) and centrifuged (10,000*g*, 20* min*, 4°C). Multiplexed magnetic bead-based immunoassay kits (Catalog# MCYTMAG-70K-P X 32, Millipore Sigma, Burlington MA) were run to evaluate tumor cytokine expression, according to the manufacturer’s instructions. Analytes assessed were granulocyte colony-stimulating factor (G-CSF), macrophage colony-stimulating factor (M-CSF), granulocyte-macrophage colony-stimulating factor (GM-CSF), vascular endothelial factor (VEGF), chemokine C-X-C motif ligand 10 (CXCL10) or interferon gamma-induced protein-10 (CXCL10/IP-10), keratinocyte-derived chemokine (CXCL1/KC), leukemia inhibitory factor (LIF), lipopolysaccharide-induced CXC chemokine (CXCL5/LIX), chemokine C-C motif ligand 2 or monocyte chemoattractant protein 1 (CCL2/MCP-1), macrophage colony-stimulating factor (M-CSF), monokine induced by γ-interferon (CXCL9/MIG), macrophage inhibitory protein-1α (CCL3/MIP-1α), macrophage inhibitory protein-1α (CCL4/MIP-1β), macrophage inhibitory protein-2 (CXCL2/MIP-2), regulated upon activation, normal T cell expressed and presumably secreted (CCL5/RANTES), tumor necrosis factor (TNF-α), interferon-γ (IFN-γ), and interleukin (IL)-1α, IL-1β, IL-2, IL-4, IL-6, IL-7, IL-9, IL-10, IL-12p40, IL-12p70, IL-13, IL-15, and IL-17.

### Quantitative Immunostaining of Tumor Progression Markers and Infiltrating Leukocytes

#### Tumor Progression and Leukocytes Markers

Tumor cell growth, apoptosis, and vascularization were evaluated with quantitative immunofluorescence staining for Ki-67, CD95 (apoptosis antigen 1), and endothelial cell-specific vascular marker, CD31, respectively, as prognostic/predictive markers for PCa progression (41–43). F4/80CD8a, and CD4 with or without FoxP3 antibodies were used to evaluate immune cell tumor infiltration (see **Table 1** for detailed antibody information and dilutions).

**Table 1:** Primary antibodies for immunohistochemical staining in orthotopic RM-9 tumors.

1° Antibody	Clone	Isotype	Immunogen	Host	Dilution	Supplier
Ki67	PA5-19462	Rabbit lgG	Human residues 1200-1300	rat	1:100	Invitrogen
CD95 (Fas)	SolA15	Rat lgG2a, κ	Recombinant protein epitope	rabbit	1:200	Millipore/Sigma
CD31 (PeCAM-1)	—	Goat lgG	Mouse myeloma cell line	goat	1:100	R&D Systems
F4/80	(clone CI:A3-1)	lgG2b	Mouse F4/80 antigen	rat	1:100	AdD Serotec
CD4	(L3T4; Rat (LOU,) clone H129.19)	lgG2a, κ	A.TH mouse CTL clone A15.17	rat	1:50	BD Bioscience
CD8a	(Lyt-2; Rat LOU, clone 53-6.7)	lgG2a, κ	Mouse thymus/spleen cells	rat	1:50	BD Bioscience
FoxP3	(clone FJK-16s)	lgG	Amino acid sequence 75-125	rabbit	1:50	Invitrogen

#### Tissue Preparation and Immunostaining

Orthotopic prostate tumors were isolated, dissected, and weighed. A portion of the tumor was placed in a 1.5-ml microfuge tube; the remainder was flash frozen in liquid nitrogen and stored in a -80°C freezer. Frozen tissue was mounted in embedding medium and sectioned at 6 μm using a cryostat (Leica CM 1900, Leica Microsystems Inc., Buffalo Grove, IL) set at -20°C. Tissue sections were taken starting at mid-tumor so that the cross-sections were closely matched between samples and representative of intratumoral tissue. Cut sections were thaw-mounted onto charged slides (Surgipath Medical Industries, Richmond, IL), and stored at -20°C.

For immunohistochemical staining, slide-mounted tissues were rinsed briefly in cold 0.15M PBS, (pH 7.2-7.4) to remove embedding medium, fixed for 10 min in acetone at -20 °C, and then rinsed in PBS (3x2 min). The slides were placed in Coplin jars containing 10% normal goat serum in PBS (30 min) to block nonspecific binding, and were rinsed in PBS (3x2 min). Slides were removed, wiped dry around the tissue, and each section was circumscribed with generic nail polish using a 3-ml syringe with a 26-gauge needle and allowed to dry. The primary antibody (or antibodies, if double-labeled) was (were) diluted following the manufacturer’s recommendation in antibody diluent (1% Triton-X™ and 5% bovine serum albumin in PBS) (see antibody information in **Table 1**). The antibody was applied to the tissue, and slides were incubated in a humidified chamber (2 h), then rinsed in PBS (5x2 min). Next, a fluorescently-tagged secondary antibody (goat anti-rat Alexa Fluor 488 or goat anti-rabbit Alexa Fluor 555, Life Technologies, Carlsbad, CA) was diluted 1:500 with the antibody diluents and applied to the tissue. The slides were incubated in a humidified chamber (2h), and then rinsed in PBS (5x2 min), and coverslipped with Prolong™ Gold Antifade containing DAPI (4’, 6-diamidino-2-phenylindole) Mountant (Life Technologies, Carlsbad, CA). Slides were placed in the dark at room temperature overnight to dry, then stored at -20°C.

Imaging of stained tissue was carried out blinded to treatment group using an Olympus BH-2 microscope equipped with a digital camera (Optronics, Goleta, CA). Quantitative analyses were performed on images captured within the tumors, specifically avoiding peritumoral regions. All images were captured using 200X total magnification. An average of 5-6 non-overlapping fields (0.162 mm^2^) was randomly sampled in RM-9 tumors. Fields used for analyses were selected using the DAPI filter to avoid bias toward immune cell markers of interest. Immunohistochemically-stained cells in each field were enumerated using Image-Pro Plus™ Version 3.1 software (Media Cybernetics, Bethesda, MD). Cell counts from each field sampled per tumor were averaged for each subject (mean total number of positive cells per sample field), and group means ± SEM were calculated (i.e., mean of a mean) with an *n* of 6-13 mice per group.

### Statistical Analysis

For hormone and cytokine analyses, two-way ANOVA and Tukey’s post-hoc tests were used to determine statistical significance between groups. Behavioral data were analyzed with SPSS 17.0 using a mixed design ANOVA with two between-group variables (Stressed/Non-stressed and Old/Young) and one repeated measures variable (test day or pre-post treatment). Huynh-Feldt degrees of freedom controlled for assumptions of compound symmetry and sphericity due to repeated measures with more than two levels (40). Additionally, comparisons between group means were performed using Student t-tests or one-way ANOVA to evaluate differences in baseline data or age-related differences, where appropriate. For significant one-way ANOVA, Bonferroni’s posthoc testing was performed to determine significant between-group differences. Pearson’s product-moment coefficients were used to relate various variables. All data were expressed as means ± standard error. An alpha level of 0.05 was considered statistically significant. The level of statistical significance is indicated as follows: * = p<0.05, ** = p<0.01, *** = p<0.001.

## Results

### Stressed Tumor-Bearing Mice Were Cachexic and Had Greater Spleen Mass, Without Affecting Tumor Mass

**Figure 2:**
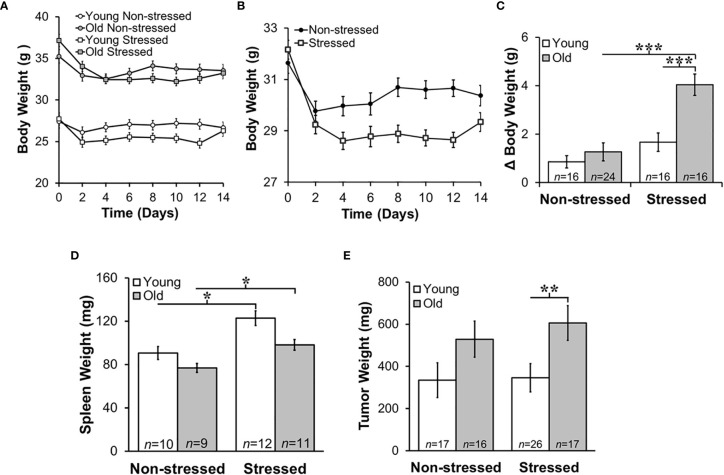
Stress Most Affected Body and Tumor Weights in Old Mice and Increased Young and Old Spleen Weight. Mean body weights ± SEM in g (*n*=16-25 per group) did not differ across time post-RM9 tumor inoculation between age-matched groups **(A)** or after collapsing data across age **(B)**. **(C)** However, there was a greater mean change (Δ) in body weight (*p*<0.001) from baseline to study endpoint in old stressed mice compared with old non-stressed or young stressed mice. **(D)** Mean spleen weights (mg) were stress-dependently increased in young and old mice (*p*<0.05). **(E)** Tumor weights (mg) were greater (*p*<0.01) in old than in young stressed mice. *p < 0.05, **p < 0.01, ***p < 0.01.

Old mice weighed significantly more than young mice (**Figure 2A**; *p*<0.001). Over the 15-day post-surgery period, non-stressed mice maintained their original body weights to a greater extent than stressed mice (**Figures 2A, B****)**. Young stressed mice weighed significantly less than young non-stressed mice on D4-12 (**Figure 2A**; *p*<0.0001). The amount of weight loss from pretreatment weights was ~5%, with an age-related difference in young and old stressed mice, and stress-related difference in old mice (**Figure 2C**; *p*<0.0001), indicating greater stress-induced cachexia in old than young mice.

Psychological stress can cause corticosterone-mediated apoptosis of spleen cells, reducing spleen weight (44). However, mean spleen weights in young and old non-stressed mice were comparable to age-matched control mice (data not shown). Moreover, spleen weight was greater in stressed than the age-matched non-stressed mice (**Figure 2D**; *, *p*<0.05). Tumors were larger in old stressed mice than young stressed mice, (**Figure 2E**; **, *p*<0.01).

### Stress Increased Anxiety in Tumor-Bearing Mice Regardless of Age

**Figure 3:**
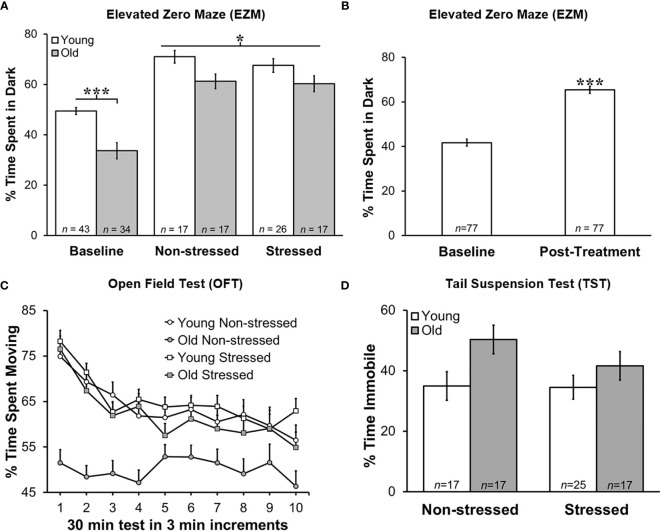
Greater Anxiety-like Behavior in Young Mice in Zero Maze, and Post-treatment than at Baseline, but No Difference in Time Immobile on Tail Suspension Test. Behavioral effects of restraint stress and isolation in young and old tumor-bearing C57BL/6 mice. **(A)** Young mice spent significantly more time in the dark post-tumor inoculation compared with old mice (*p*<0.0001). Stress did not significantly alter the time spent in the dark for either young or old mice. Young mice spent significantly more time in the dark compared with old mice overall (*p*<0.0001). **(B)** When groups were collapsed, all mice spent significantly more time in the dark post-treatment compared with baseline (*p*<0.0001). Data are expressed as the mean % of time spent in the dark ± S.E.M. **(C)** Old non-stressed mice were significantly less active throughout the test compared with old stressed mice (*p*<0.05) and with young mice regardless of being stressed or non-stressed (*p<*0.05). Data are expressed as the mean % spent moving ± S.E.M. for each treatment group for each 3 min increment over the 30 min time period for the OFT (*n*=17-26 mice per group). **(D)** There were no group differences in percent time immobile on TST. *p < 0.05, ***p < 0.001.

Baseline EZM testing revealed that young mice spent significantly more time in the dark than old mice, suggesting that young mice act more anxious than old mice based on their avoidance of the open spaces and preference for the closed dark spaces (**Figure 3A**; *p*<0.0001). All groups showed increased (*p*<0.05) anxiety-like behavior in the EZM 13 days after tumor cell inoculation (**Figure 3A**). In young non-stressed mice, serum corticosterone levels positively correlated with time spent in the dark (*r*=0.55, *p*=0.035). However, in young stressed mice, tumor progression was significantly related to: a) the amount of time animals spent in the dark during D2 of the EZM (*r*=0.508, *p*=0.013); b) the amount of learned helplessness displayed during the TST (*r*=-0.454, *p*=0.034); and c) between the amount of activity in the open field (*r*=-0.423, *p*=0.045). Tumor progression was also correlated with time spent in the dark on D2 of EZM in old non-stressed mice (*r*=0.525, *p*=0.037).

Regardless of age, non-stressed and stressed mice spent significantly more time in the dark post- than pre-tumor inoculation (baseline; **Figure 3B**). Repeated restraint and isolation stress did not alter the amount of time young or old mice spent in the dark compared with young or old non-stressed groups. When data for young and old mice were collapsed across baseline and across tumor inoculation groups, all mice spent significantly more time in the dark (*p*<0.0001) after developing tumors than before tumor inoculation (**Figure 3B**).

Tumor weight correlated with the percent time spent in the dark for all groups except old stressed mice (young non-stressed: *r*=-0.55, *p*<0.035; young stressed: *r*=0.51, *p*<0.013; old non-stressed: *r*=0.53, *p*<0.037). In addition, there was a significant positive relationship between corticosterone levels post-treatment, and how much learned helplessness an animal demonstrated during the TST (*r*=0.512, *p*=0.018). Older non-stressed mice displayed greater activity levels in both behavioral tests.

### Greater Activity in the Open Field Supports Higher Anxiety in Old Stressed Mice

Old non-stressed mice were significantly less active throughout the OFT than old stressed mice or young non-stressed and stressed mice (**Figure 3C**; *p*<0.05). No significant differences were found for age or groups regarding the time spent in the perimeter or center of the open field (data not shown). Tumor weight in each treatment group was negatively correlated with the amount of activity in the open field (e.g., larger tumors were associated with less activity; young non-stressed: *r*=-0.45, *p*<0.045; young stressed: *r*=-0.42, *p*<0.045; old non-stressed: *r*=-0.595, *p*<0.12; old stressed: *r*=-0.49, *p*<0.044).

### Old Mice Exhibited More Depressed-Like Behavior

Old mice demonstrated more depression-like learned helplessness behavior than young mice during the TST *p*<0.02). Stress status did not influence depression-like behavior in either age group **(****Figure 3D****)**. In young stressed mice, tumor weight (*r*=0.454, *p*<0.034) and serum corticosterone (*r*=0.51, *p*<0.02) was associated with depression-like behavior.

### HPA Activity Increased Post-Treatment When Data Is Collapsed for Age

**Figure 4:**
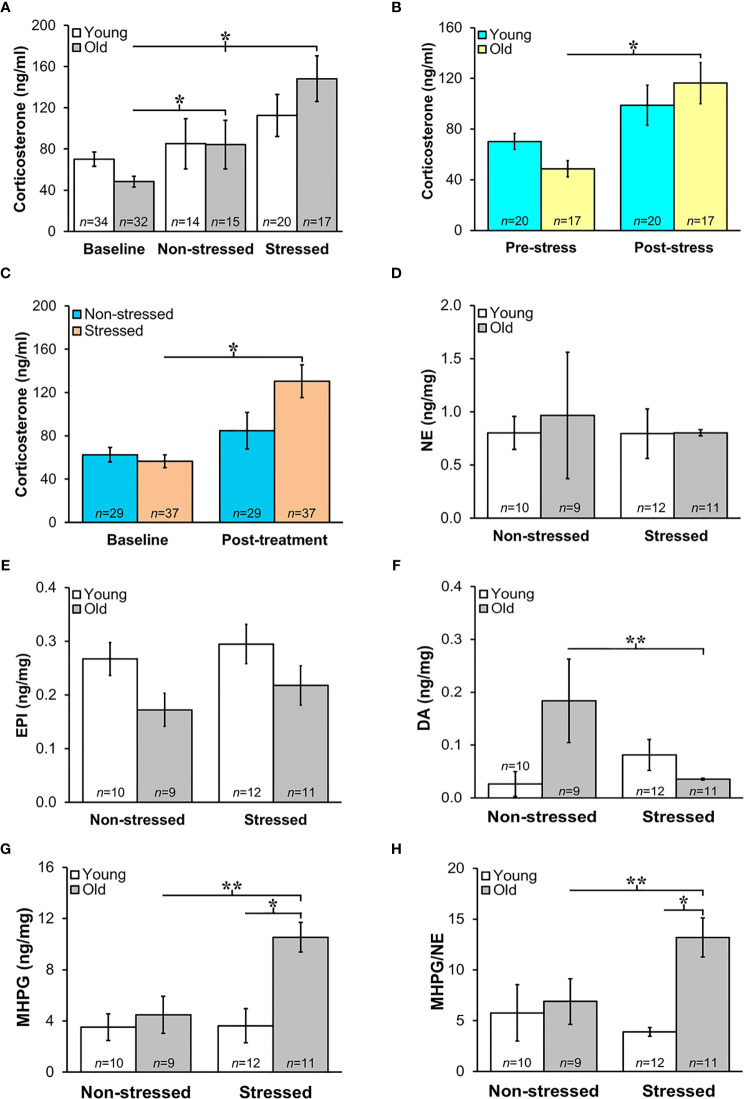
**(A)** Increased HPA and SNS Activation in Stressed Old Mice. Mean circulating corticosterone levels (ng/ml ± S.E.M) from young and old non-stressed and stressed mice at baseline and D15 post-tumor inoculation. Old stressed mice had a significant increase in corticosterone levels post-treatment than at baseline. **(B)** After data are collapsed across age, Stressed mice had significantly higher corticosterone levels than Non-stressed mice (**p < 0.02). Data are expressed as corticosterone in ng/ml ± S.E.M. *Daily restraint stress began1 day after tumor inoculation. **(C)** Corticosterone expressed in ng/ml was higher (*p < 0.05) post-treatment compared with baseline levels in stressed compared with non-stressed mice. **(D, E)** Tumor expression of NE and EPI (ng/mg) were similar in all treatment groups. **(F, G)** However, in old mice, prostate dopamine, a precursor in NE synthesis, was lower (**p < 0.01) in stressed than non-stressed mice, and prostate MHPG, a metabolite of NE degradation, was elevated in old stressed mice compared with old non-stressed or young stressed mice (**p < 0.01 or *p < 0.05, respectively). **(H)** Likewise, MHPG/NE ratios, used to estimate NE turnover, was also higher in prostate tumors from old stressed mice than from old non-stressed or young stressed mice (**p < 0.01 or *p < 0.05, respectively). *p < 0.05, **p < 0.01.

Prior to tumor inoculation, old mice had lower baseline corticosterone levels than young mice (**Figures 4A, B**; *p*<0.02). A one-way ANOVA to assess treatment differences in 18-month-old mice revealed that repeated restraint and isolation stress significantly increased corticosterone levels (**Figure 4A**; *p*<0.04). In young stressed and non-stressed mice, corticosterone levels were not significantly different compared with baseline levels prior to tumor inoculation. Although not significant, there was a trend for old mice to display increased corticosterone levels compared with young mice post-tumor inoculation. When corticosterone levels were collapsed for stressed and non-stressed mice across age and compared to baseline values, there was a positive interaction between pre-post tumor inoculation and treatment group (*p*<0.02). Stressed mice had increased corticosterone levels compared with non-stressed mice (**Figure 4B**; *p*<0.05). However, old, but not young, stressed mice (**Figure 4C**) had significantly increased serum corticosterone levels compared with old non-stressed mice post-treatment (*p*<0.04). RM-9 tumor weight was correlated with circulating corticosterone levels in young and old stressed and old non-stressed mice (young stressed: *r*=0.73, *p*<0.005; old non-stressed: *r*=0.65, *p*<0.001; old stressed: *r*=0.43, *p*<0.01).

### Sympathetic Nerve Activity in Prostate Tumors Increased in Old Stressed Mice

Mean prostate norepinephrine and epinephrine concentrations (**Figures 4D, E**, respectively) remained stable in young and old non-stressed and stressed mice. However, in old mice, the mean prostate concentration of the norepinephrine precursor, dopamine, was lower in stressed than in non-stressed mice (**Figure 4F**; *p*<0.01). In contrast, stress in old mice more than doubled the prostate levels of the norepinephrine catabolite, MHPG, compared with age-matched non-stressed mice (**Figure 4G**; *p*<0.01). Likewise, in old mice, MHPG/norepinephrine ratio, an indicator of norepinephrine turnover, was greater (**Figure 4H**; *p*<0.01) in stressed than non-stressed mice; this ratio was also greater in old than young stressed mice (**Figure 4H**; *p*<0.05).

### Proliferation, Apoptosis and Endothelial Cell Markers Support Positive Effects of Stress RM-9 Tumor Progression

**Figure 5:**
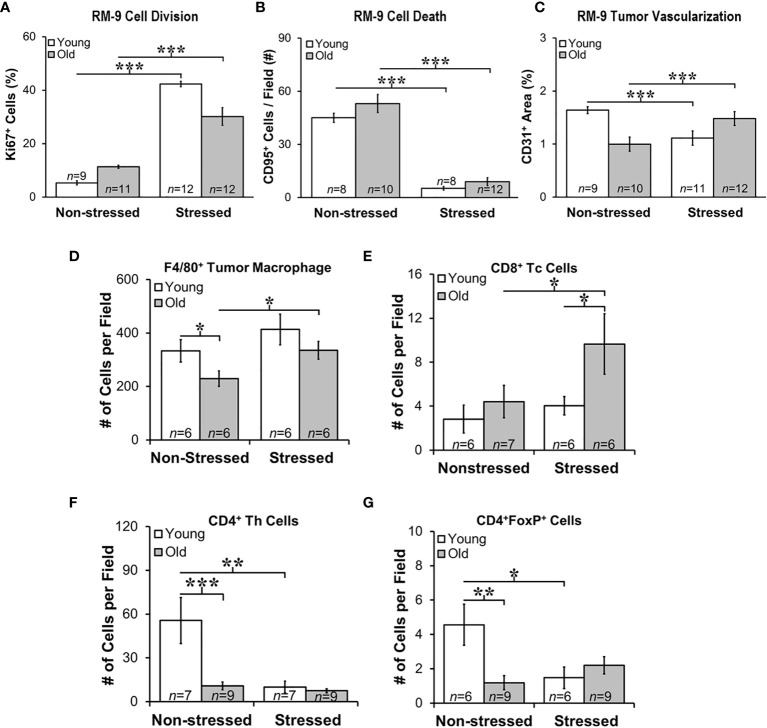
Stress Promoted Tumor Proliferation and Apoptosis, but Age-dependent Infiltration of Immune Cell Subsets and Vascularization Support Age-dependent Anti-tumor Defense Mechanisms. Quantitation of immunostaining for proliferation **(A)**, apoptosis **(B)**, angiogenesis **(C)**, F4/80^+^ macrophages, and CD8^+^, CD4^+^, and CD4^+^FoxP^+^ cells **(D–G**, respectively) in young (open bar) and old (gray bar) non-stressed and stressed mice D14 after orthotopic prostate tumor inoculation. **(A)** Stress increased prostate tumor expression of Ki67 in young and old mice compared with non-stress age-matched mice. **(B)** Conversely, CD95 expression was lower in RM-9 tumors from stressed than in non-stressed young and old mice. **(C)** CD31 immunoreactivity was higher in stressed than non-stressed old mice, but in young mice expression was greater in non-stressed than the stressed group. **(D)** F4/80^+^ tumor macrophages were fewer (*p < 0.05) in old than young non-stressed mice, but were higher in stressed than non-stressed old mice. **(E)** CD8^+^ Tc cell numbers were similar in non-stressed mice, but were higher in old than young in stressed mice. Moreover, more (*p*<0.05) CD8^+^ cells were present in old than young stressed mice. **(F)** CD4^+^, including those expressing FoxP3^+^ **(G)**, were lower in old than young non-stress mice, and in young stressed than non-stressed mice. *p < 0.05, **p < 0.01, ***p < 0.001.

Quantitative immunofluorescence staining in RM9 tumors for proliferation (Ki-67), apoptosis antigen 1 (CD95), and microvessel density (CD31) markers (**Figures 5A–C**, respectively), support a tumor-promoting effect of restraint stress in young and old mice. Stress dramatically increased the percentage of Ki-67^+^ cells in RM-9 tumors in both young and old mice (*p*<0.001; **Figure 5A**). In contrast, stress reduced expression of CD95 in RM-9 tumors from young and old stressed mice (*p*<0.001; **Figure 5B**), consistent with tumor cell shedding of this ligand to escape immune-mediated apoptotic cell death. The mean number of CD95^+^ cells per field was similar in young and old non-stressed mice, about ~50-60 per field (**Figure 5B**). Expression of CD31 was lower (*p*<0.001; **Figure 5C**) in prostate tumors from stressed than non-stressed young mice, but CD31 expression was conversely higher (*p*<0.001) in prostate tumors from stressed than in non-stressed old mice.

### Stress Differentially Altered Immune Cell Infiltration into Tumors in Young and Old Mice

Myeloid and T cells are crucial components of the immune response to cancer, as they play major roles in PCa initiation and progression (42). Tumor-associated macrophages (TAMs) are well represented in PCa, their presence in murine models promotes tumor progression, and their presence generally strongly correlates with poor prognosis. T cell infiltration in tumors is essential for the immunologic response to tumor tissue. Therefore, we evaluated immune cell infiltrates in RM-9 tumors. F4/80^+^ macrophages, and CD4, CD4/FoxP3^+^ and CD8^+^ T cells revealed tumor infiltration of immune cells into RM-9 tumors as shown in **Figures 5D–G****)**.

Most of the leukocyte infiltrates into the tumors were macrophages (**Figures 5D**). There were main effects of age (*p*=0.041) and stress (*p*=0.036), but no significant interaction between age and treatment for intratumoral F4/80^+^ macrophage number (**Figure 5D**). There were fewer F4/80^+^ macrophages in RM-9 tumors from old compared with young non-stressed groups (*p*<0.05). In contrast, tumors from old stressed mice had more (*p*<0.05) F4/80^+^ macrophages than in non-stressed controls (**Figures 5D**) (*p*<0.02). There were main effects of age (*p*<0.03) and stress (*p*<0.05) for tumor CD8^+^ Tc cells, which were sparse and widely dispersed (data not shown). In old mice, stress increased (*p*=0.05) tumor CD8^+^ T cell numbers, and they were more abundant (*p*=0.05) in old than young stressed mice (**Figure 5E**). CD8+ Tc cells in RM9 tumors were more numerous (p<0.05) in old than young stressed mice, and in stressed mice were greater (p<0.05) in old than young mice (**Figure 5E**). In contrast, tumor CD4+ Th cells were most abundant in young non-stressed mice (**Figure 5F**), but were significantly reduced (p<0.01) by stress. Tumor CD4+ Th cells were lower (p<0.001) in old than young non-stressed mice (**Figure 5F**).

CD4^+^FoxP3^+^ T cells were sparse in RM-9 tumors (**Figure 5G**). Two-way ANOVA revealed a significant interaction between age and stress status (*p*=0.02) in tumor CD4^+^FoxP3^+^ cells number (**Figure 5F**). In young mice, stress reduced tumor CD4^+^FoxP3^+^ cell infiltration (*p*<0.03). Fewer CD4^+^FoxP3^+^ cells infiltrated tumors from old than from young non-stressed mice. Unlike young mice, there was no effect of stress on CD4^+^FoxP3^+^ cell tumor infiltration in old mice (**Figure 5G**).

### Growth Factors and Chemokine Expression in Prostate Tumors Were Age- and/or Stress-Dependent

**Figure 6:**
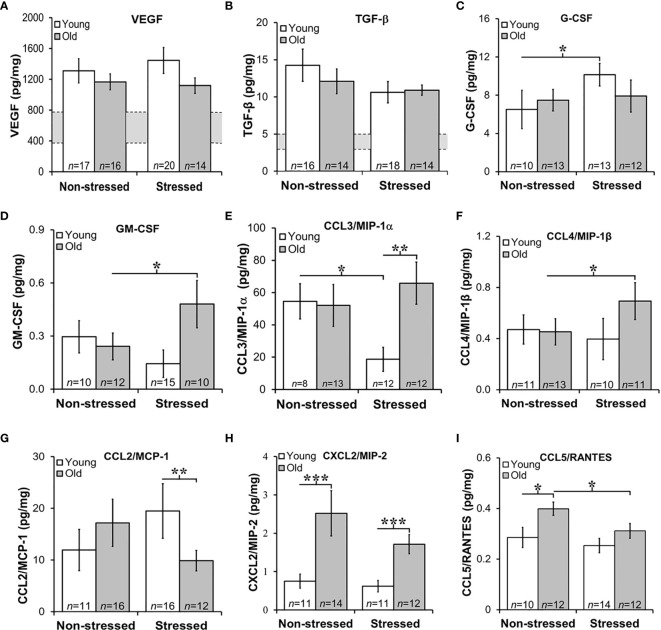
Growth Factors and Chemokines Expression in Prostate Tumors. **(A, B)** VEGF and TGF-β were highly expressed in RM-9 tumors, regardless of age or treatment group. The gray bar indicates the range of VEGF **(A)** and TGF-β **(B)** expression in the murine prostate gland in the absence of RM9 tumors (based on collapsed data from an n of 8 per age group of normal prostate samples; no age-related differences were identified in these control samples). Mean tumor expression of VEGF and TGF-β expression were higher than in basal prostate levels, but no age- or stress-related differences were uncovered. **(C)** In young mice, RM-9 tumor G-CSF levels were higher (*p < 0.05) in stressed than non-stressed mice. **(D)** In contrast, tumor GM-CSF in old mice was greater (*p < 0.05) in stressed than non-stressed mice. **(E)** CCL3/MIP-1α was one of the most highly expressed chemokine that was quantified, and one or two chemokines where effects of both age and stress were identified. In young mice, stress reduced (*p < 0.05) tumor CCL3/MIP-1α compared with levels in non-stress mice. In tumors from stress mice, CCL3/MIP-1α was higher (**p < 0.01) in old than young mice. **(F)** An increase (*p < 0.05) in tumor CCL4/MIP-1β levels was observed in old compared with young stressed mice. **(G)** In the stressed groups, CCL2/MCP-1 was lower (**p < 0.01) in old than young mice. **(H)** Only effects of age were observed for CXCL2/MIP-2 such that levels were higher in old than young mice regardless of stressed group (***p < 0.001). **(I)** Tumor CCL5/RANTES concentrations were reduced in old compared with young stressed mice, but in non-stressed mice, levels were higher in old than in young mice.

Tumor growth and progression are influenced in a complex way by a multitude of growth factors and cytokines in the prostate microenvironment. Here, we evaluated tumor expression of growth factors and cytokines prognostic for PCa progression and/or poorer survival. Additionally, we assessed how stress and age influences expression of cytokine/chemokines that may be influenced by age and/or stress and hence serves as novel markers for age- and/or stress-related tumor progression. VEGF was used to assess angiogenesis (45), whereas TGF-β and IL-6 can serve as tumor promoters (46) that induce VEGF to regulate prostate growth (45, 47), and promote Th2 cell-mediated humoral immunity (48). Stress hormones can regulate both growth factors in PCa models (49). In our orthotopic PCa model, VEGF and TGF-β were highly expressed in prostate tumors, with similar levels regardless of age and treatment group **(****Figures 6A, B**, respectively). Tumor VEGF and TGF-β concentrations were higher in all mice than non-treated control mice (indicated for VEGF and TGF-β by the gray horizontal bar; **Figures 6A, B****)**, but were comparable across age and treatment group. (The gray horizontal bars in **Figures 6A, B** represent the mean cytokine levels ± SD; *N*=10 untreated young and old C57Bl/6 mice).

GM-CSF is a potent cytokine with anti-tumor activity that works by inducing expression of TNF-α and IL-1 (50). In contrast, G-CSF is a poor prognosticator in human PCa as it promotes PCa development *via* neurogenic influence on autonomic nerves (51). G-CSF modulates the growth/sprouting and survival of sympathetic nerves, which promotes PCa growth and dissemination in metastatic models. RM-9 prostate tumors expressed both G-CSF and GM-CSF (**Figures 6C, D**, respectively); however, tumor expression of poor survival marker, G-CSF was ten-fold higher than GM-CSF. Restraint stress increased (*, *p*<0.05) tumor G-CSF in young mice (**Figure 6C**), but stress increased (*, *p*<0.05) tumor GM-CSF in old mice (**Figure 6D**), respectively.

Several chemokines that can promote tumor growth, angiogenesis and metastases were expressed in RM-9 prostate tumors (**Figures 6E–I**) (52, 53). From highest to lowest concentration they were MIP-1α > MCP-1 > MIP-2 > MIP-1α = or > RANTES. Effects of stress were found for CCL3/MIP-1α (**Figure 6E**) and CCL4/MIP-1β (**Figure 6F**). Among the greatest expressed chemokines of the five evaluated, were CCL3/MIP-1α and CCL2/MCP-1. CCL3/MIP-1α was expressed at high levels in non-stressed young and old and stressed old mice. Interestingly, CCL3/MIP-1α, a cytokine marker of poor prognosis (53), was reduced by about half in young stressed mice compared with both young non-stressed (**, *p*<0.01) and stressed old mice (*p*<0.01). Surprisingly, stress failed to reduce this chemokine in old mice. Expression of CCL4/MIP-1β was about ten-fold lower than CCL3/MIP-1α. Also in contrast to CCL3/MIP-1α, significant differences were observed only in tumors from old stressed mice, which had higher tumor CCL4/MIP-1β levels than tumors in young non-stressed mice (*p*<0.05). Although tumor CCL4/MIP-1β expression in old stressed mice were greater than in young stressed mice, this finding did not reach statistical significance.

Largely tumor derived, CCL2/MCP-1 exerts autocrine-mediated promotion of tumor growth and invasion (53, 54), and paracrine-mediated recruitment of myeloid-derived suppressor cells (MDSCs) that enhance tumor cell survival (55, 56). CCL2/MCP-1 is expressed in prostatic stromal cells, where it stimulates prostatic epithelial cells growth. In this study, age-related differences were uncovered for CCL2/MCP-1 and CXCL2/MIP-2. Tumor levels of CCL2/MCP-1 were lower in old than young stressed mice. Tumor-derived CCL2/MCP-1 produced in tumors in this study ranged from ~10 to 20 pg/ml). CCL2/MCP-1 was ~2-fold lower in old than young stressed mice (**Figure 6G**: **, *p*<0.01). No differences in this chemokine were observed between non-stressed and stressed mice.

**Figure 7:**
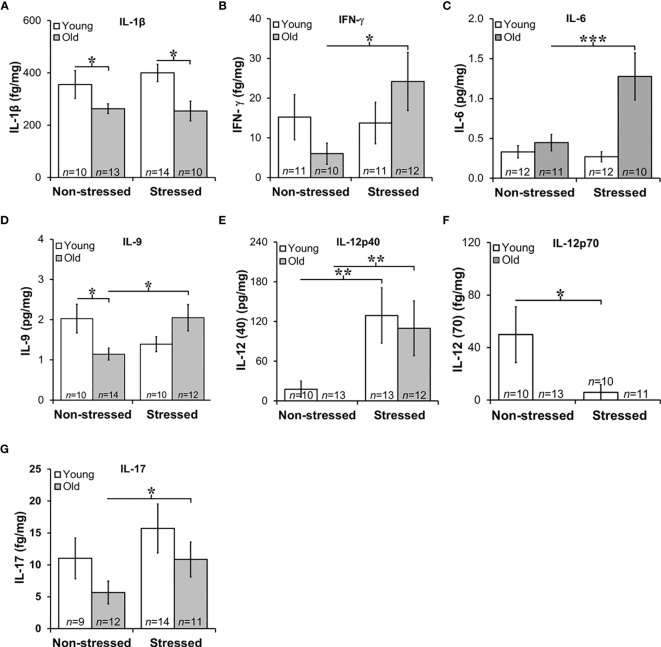
Expression of Interleukins in Prostate Tumors. **(A)** IL-1β expression was lower (p<0.05) in old than in young tumors, regardless of the stress-related group. **(B, C)** There was an effect of stress on IFN-γ and IL-6 tumor expression in old mice such that stress mice had elevated tumor expression than non-stressed mice (***p < 0.001). **(D)** Tumors from old stressed mice expressed higher levels of IL-9 than young stressed mice, but in non-stressed mice, tumor IL-9 levels were lower in old than young mice. **(E)** Tumor levels of IL-12p40 were low in non-stressed mice, regardless of age. In both young and old mice stress significantly increased (**p < 0.01) tumor IL-12(p40) expression compared with non-stress age-matched mice **(F)** Overall, IL-12p70 expression in prostate tumors was lower than IL-12p40 shown in **(E)**, with low levels in old mice regardless of stress group. Still, in young and old mice, psychosocial stress reduced (**p < 0.01) tumor IL-12p70 expression. **(G)** Prostate tumor IL-17 levels were low in all treatment groups, but a stress-related increase (*p < 0.05) in tumor levels of IL-17 was observed in old compared with young mice.

CXCL2/MIP-2 ranged from ~ 0.75 to 2.5 pg/mg tumor tissue. In both stressed and non-stressed, old mice expressed higher levels of CXCL2/MIP-2 than young mice (**Figure 6H**: *, *p*<0.05), supporting an age-related increase of this chemokine. Secreted by endothelial cells, CCL5/RANTES induces autophagy in PCa cell lines (57). Low levels of CCL5/RANTES were produced by RM-9 tumors across all treatment groups. Both age and stress effects were demonstrated for tumor expression of CCL5/RANTES (**Figure 6I**: *, *p*<0.05). Tumors from old non-stressed mice had higher levels of CCL5/RANTES (*, *p*<0.05) than young non-stressed mice. In stressed mice, CCL5/RANTES reduced (*, *p*<0.05) in old compared with young mice. No difference in CCL5/RANTES levels was observed between non-stressed and stressed young mice or between young and old stressed mice. CXCL10/IP10, a negative regulator of tumor growth and promoter of CD8^+^ T cell tumor infiltration in human prostate LNCaP cells (56), was expressed at comparable levels (~6 pg/mg) in all treatment groups (data not shown), despite low tumor levels of its chief inducer, IFN-γ (fg/mg; see **Figure 7B**). There were no effects of age or stress on CXCL10/IP-10 (data not shown).

### Stress Enhanced Tumor IL-6, IL-9, 12p40 and IL-17, and Suppressed IL-12p70 in Old Mice

In the aged microenvironment, the transition of the prostate to a hyperplastic state is characterized by inflammatory infiltrates with distinct immune cytokine-secreting signatures that support immune cell polarization toward unfavorable disease outcomes (58). To assess age and stress effects on the tumor cytokine milieu, multiplex antibody-based affinity protein cytokine arrays were used. The cytokine milieu was consistent with immunohistochemical observations of increased macrophages and T cell infiltration.

Chronic low-level production of TNF-α in the tumor microenvironment is a hallmark feature of PCa (59), and is consistent with low TNF-α expression in RM-9 tumors and no effects of age or stress (data not shown). Elevated IL-1β expression has been implicated in human prostate pathology, particularly linked to human prostatic proliferative inflammatory atrophy (60). In RM-9 prostate tumors, IL-1β expression was low in RM-9 tumors in young and old mice (**Figure 7A**). Still, IL-1β was significantly reduced in old compared with young non-stress and stressed mice (**Figure 7A**). IL-1β-induced IFN-γ from activated immune cells sensitizes PCa cells toward Fas-mediated cell death (61), but is also reported to induce immune escape by neuroendocrine-like differentiation in human prostate basal-epithelial cells (62). IFN-γ can upregulate MHC class I expression and induce Th1 cell polarization, driving anti-tumor-specific immune responses mediated in part *via* IL-12 (63). Consistent with the low IL-1β production within the tumors, IFN-γ was suppressed in RM-9 tumors. However, IFN-γ was significantly elevated in the prostate tumors of stressed old mice (**Figure 7B**; *, *p*<0.05).

Several interleukins expressed in RM-9 tumors were affected by stress. IL-6 is associated with an aggressive PCa phenotype by inducing VEGF, promoting tumor cell proliferation, suppressing apoptosis, and driving Th cell differentiation of regulatory Th cells (64). IL-9 has both anti- and pro-tumor actions that are not well understood (65), and IL-9 has complicated anti-tumor and pro-tumor properties mediated by innate and adaptive immunity (66), including in PCa (67). In RM-9 tumors from stressed groups, IL-6, and IL-9 expression was greater in old than in young mice (**Figures 7C, D**, respectively; **Figure 7C**: *, *p*<0.05; **Figure 7D**: *, *p*<0.05). Additionally, IL-9 levels were significantly less in old non-stressed mice than young non-stressed mice, suggesting an age- and stress-related difference in production of this cytokine.

Primarily produced by activated myeloid cells, IL-10 and IL-12 play immunoregulatory roles in host defense and immune homeostasis, typically with opposing immune suppressive and enhancing actions, respectively. IL-10 induces anti-tumor actions by inhibiting angiogenesis and cell proliferation of PCa cells (68). PCa expression of IL-10 (mean levels ~0.06 pg/mg) was not affected by age or stress, (data not shown). Two forms of IL-12 were expressed in RM-9 tumors, prostate tumor-promoting IL-12p40 and tumor growth suppressing IL-12p70 (63) (**Figures 7E, F**, respectively). RM-9 tumor expression of the tumor promoting IL-12p40 was greater in stressed young and old mice than age-matched non-stressed mice (**Figure 7E**: *, *p*<0.05). The tumor suppressor, IL-12p70 was below the level of detection in old mice regardless of whether these mice were stressed or not stressed. IL-12p70 was detectible in tumors from young mice. IL-12p70 was lower in RM-9 tumors from stressed young mice, and non-stressed young mice (**Figure 7F**: *, *p*<0.05).

In several murine models, IL-17 is reported to promote PCa progression (69). In this study, IL-17 expression in RM9 tumor was low (fg/mg range) regardless of age or stress, but stress significantly increased tumor expression of IL-17 in old, but not young mice (**Figure 7G**: *, *p*<0.05). No differences were observed in IL-17 between non-stressed and stressed young mice or non-stressed old and stressed young mice. IL-1α, IL-2, IL-4, IL-5, and IL-7 were expressed in prostate tumors in the fg/mg range, but there were no effects of age or stress on these cytokines (data not shown).

## Discussion

The primary goals of this research were to establish a novel age-relevant rodent model of PCa and to identify how psychological stress interacts with the age-dependent tumor microenvironment to influence cancer progression. To achieve these goals, we repurposed a non-metastatic, orthotopic, syngeneic mouse model of PCa previously used to study the efficacy of cytokine therapies on tumor growth. To our knowledge, this is the first study to evaluate interactions between age and mild repeated acute psychosocial stress in PCa. Stress age-dependently influenced the immune response in RM-9 tumors. The intratumoral immune response differed through its suppressive effect on the Th1-type cytokine production from CD4^+^ T cells; stress-increased tumor IL-12p40; this effect was consistent across age. This study is the first to report the significant effects of age and psychosocial stress on PCa progression in an orthotopic mouse model. PCa is largely a disease of older men, and age often plays a role in treatment choice. In fact, the most common high-risk factor for PCa and lower overall survival is age. Our findings provide support for age-related pathophysiologic differences in RM-9 tumors. Moreover, they underscore the use of age-appropriate animal models to better understand the clinical effects of stress and their consequences for the efficacy of anti-cancer drugs than young adult rodent cancer models.

In this murine prostate model, stressed mice weighed less than non-stressed mice, supporting stress-promoting cachexia, as defined as >5% weight loss; this effect was greater in old than in young mice (~10% vs. ~7%, respectively). This finding is consistent with reports in other rodent models of PCa (70, 71) and breast cancer (27), which in the latter was reversed by G-CSF inhibition (72). The stress-induced splenomegaly in the present report is consistent with tumor detection and systemic age-dependent tumor-host interaction that can influence host defense against tumor development.

We found that restraint- and isolation-induced stress increased the activity of the SNS (based on mean MHPG/NE ratios) and HPA axis to a greater degree in old than young mice, negatively affected behavior, and promoted tumor progression in an age-dependent manner. Although RM-9 prostate tumor growth, as assessed by tumor weight, was not significant, more refined assessments of tumor status (cell proliferation/death) and cytokine expression supports that isolation/restraint stress increased RM-9 cell proliferation and reduced apoptosis regardless of age. Moreover, our findings indicate opposing age-dependent effects of moderate repeated psychosocial stress on angiogenesis, based on stress-mediated suppression and enhancement of angiogenesis in young and old mice, respectively. Overall, our findings of an exaggerated increase in SNS activity in old stressed mice are consistent with Hassan et al. (22, 73), who demonstrated that stress accelerated PCa development in another murine PCa model that was mediated *via* increased activity of the SNS.

Our findings also indicate that chronologic age moderated the effects of combined isolation and restraint stress on the infiltration of leukocyte subsets important for tumor immunity. Surprisingly, all mice displayed increased anxiety-like behaviors from baseline measures. One interpretation of this finding is that surgical stress from tumor inoculation increased anxiety-like behavior, and that there was no additive effect of isolation combined with restraint on this measure. However, we cannot exclude the possibility that the mice simply learned that there was nothing to be gained in exploration of the open and brightly-lit quadrants. However, we cannot discount that tumor presence (possibly *via* cytokines entering the circulation) induced or influenced a stress response. Cytokines released during the immune response can alter both physiology and behavior [reviewed in (74)], and mouse behavior can also change in the presence of tumor development (75). Previous studies with mice have shown that the presence of subcutaneous RM-9 tumors can significantly alter the profile of secreted cytokines (76).

In the present experiment, larger tumors were correlated with increased anxiety-like behaviors. Older men with PCa can experience both cancer-related physical and psychological stress (77). Moreover, stress reduction techniques can minimize the negative effects of both physical and cognitive reactions to stress (78). Thus, the link between tumor presence and stress is likely to be clinically important. Notably, despite increased post-inoculation anxiety-like behavior in all groups, stressed mice exhibited more corticosterone and anxiety-like behaviors than non-stressed mice, indicating that the isolation and restraint successfully produced non-tumor-related changes possibly detrimental to cancer recovery.

There were also important age-related differences in response to stress. Old mice had lower baseline levels of circulating corticosterone than young mice. Although corticosterone levels did not differ between young and old non-stressed mice with PCa. Given the lower baseline corticosterone levels, old mice had a greater increase in corticosterone levels than young mice. Similarly, old stressed mice with PCa had a greater change in corticosterone levels than the young stressed mice with PCa. These findings support that although old mice started at a lower level of circulating corticosterone, they had a greater stress response compared with young mice.

Old stressed mice were significantly more active in a novel environment than old non-stressed mice. In fact, old stressed mice were as active as young mice, in which stress did not change activity levels. This suggests that in old mice stress prevented the normal decline in activity, but did not affect overall activity levels in young mice. These findings confirm previous research that old mice can display increased locomotion and exploratory behavior when exposed to a stressor (79). In addition, old mice demonstrated greater depression-like behaviors in an inescapable learned helplessness situation. Old mice gave up struggling on the TST much sooner than young mice. Anxiety and depressive behaviors are often co-morbid; indeed, one predictor for cancer-related depression risk is the presence of anxiety disorders (80). Studying the dynamic interplay of these behaviors, especially in the context of age, can increase our understanding of how individuals from different age groups respond to negative news, such as a cancer diagnosis and the subsequent effects this response might have on the patient’s prognosis. The dynamic interplay between the mind and body is well known, and psychological distress has been found to be a predictor in cancer mortality (81). In fact, patients with high levels of psychological distress, including depression and anxiety, have a 27% increase in cancer mortality (82).

**Figure 8:**
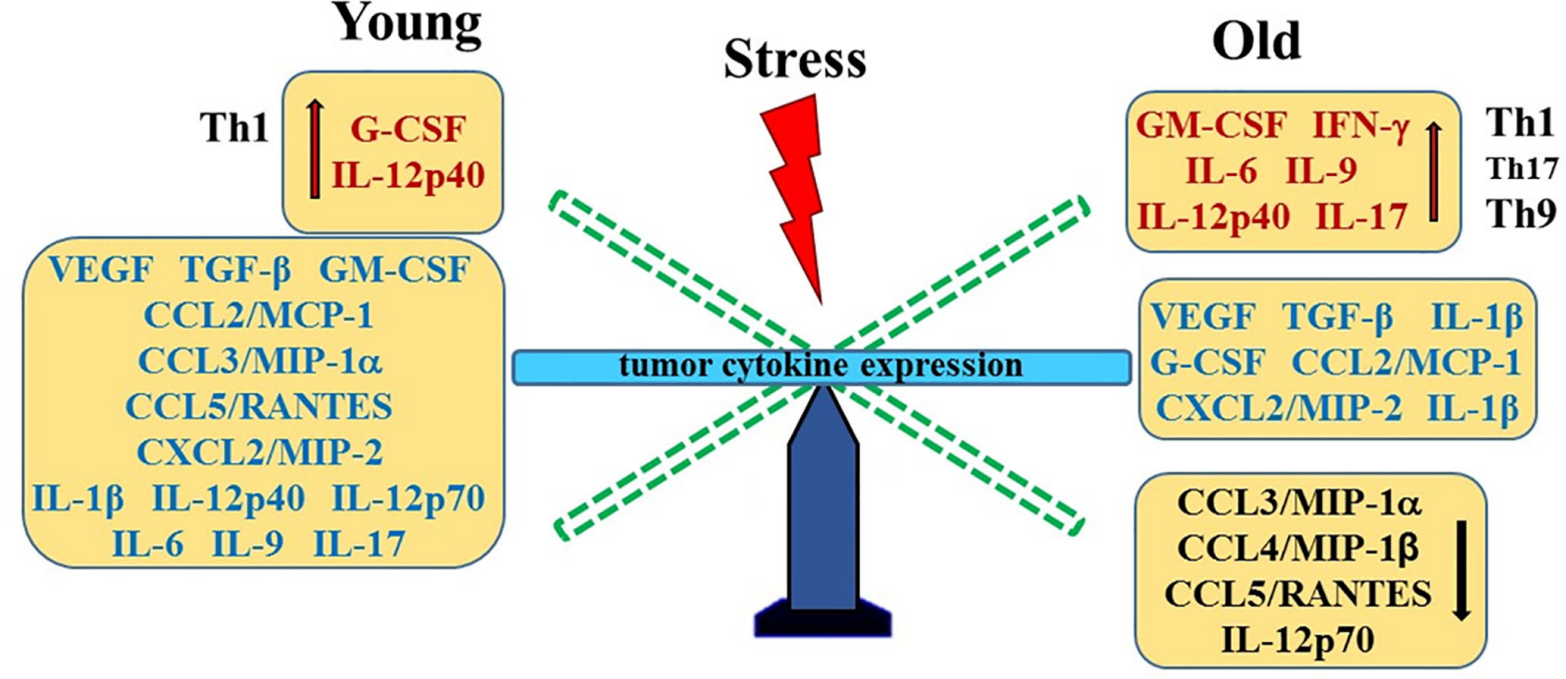
Stress- and Age-Mediated Changes in Prostate Cytokine Expression. A summary of the changes in tumor chemokine/cytokine expression in young (left) and old (right) stressed mice is represented as a “teeter-totter’ with an increase or decrease in expression represented as an elevated or depressed bars, respectively. CCL, C-C Motif Chemokine Ligand; CXCL, (C-X-C motif) ligand 1; G-CSF, Granulocyte Colony-stimulating Factor; GM-CSF, Granulocyte/Monocyte Colony-stimulating Factor; IFN, interferon; IL, interleukin; MCP, Monocyte Chemoattractant Protein-1; MIP, Macrophage Inflammatory Protein; RANTES, Regulated upon Activation, Normal T Cell Expressed and Presumably Secreted; TGF, Transforming Growth Factor; Th, T-helper; VEGF, Vascular Endothelial Growth Factor (VEGF).

Our data infers that age also appears to be a factor in tumor size. RM-9 tumors grew faster in old mice than in young mice. A study by Reed and colleagues (83) showed that subcutaneously injected prostate TRAMP-C2 tumor cells grow at a similar rate in 20-month-old as in 4-month-old syngeneic mice. The lack of age-related difference in tumor growth between these models may be due to differences in subcutaneous compared with orthotopic placement of tumor cells. Age-related differences in tumor growth rates appear to be tumor type-specific. Whereas the growth of some tumors is slower in old animals (84, 85), certain tumors, e.g. some sarcomas, melanoma and liver cancer, grow more rapidly in older animals. However, no age-related differences were observed in the growth of melanoma and AKR lymphoma cells (84–86). Age-related differences have been linked with age-associated decline in anti-tumor immunity, different immunogenic properties and the tumor microenvironment of different types of tumors. Factors shown to be contributory to age-related differences in tumor progression include the local microenvironment, angiogenesis, apoptosis, inflammation, and host anti-tumor immunity (84–86). Greater expression of several chemokines may contribute to the age-related increase in tumor growth in old compared with young stressed mice. A summary of age- and stress-related changes in prostate tumor cytokines is illustrated in **Figure 8**.

Our finding of higher concentrations of CCL3/MIP-1α, CCL4/MIP-1β and CXCL2/MIP-2 in old versus young stressed mice are consistent with greater tumor growth in old stressed mice and poorer prognosis (87). Similarly, IL-1α and IL-9 expression were influenced by age. These findings suggest age-specific tumor immunity response profiles that can be differentially influenced by psychosocial stressors. Th1- and Th9/17-driven immune responses were observed in young and old mice, respectively. Several studies have reported that secretion of these chemokines from tumor-infiltrating macrophages promote prostate tumorigenesis *via* their effects on VEGF-mediated tumor vascularization (52, 88–90). In the present study, tumor VEGF and TGF-α1 expression were higher than in non-tumor-bearing mice, but were not affected by age or stress. In contrast, age-dependent effects of stress were demonstrated for G- and GM-CSF, which may differentially affect tumor progression in young and old mice. Age- and/or stress-related influences on tumors have implications for cancer treatment. For example, aging differences in tumor vascularization with certain types of cancer (91) could affect therapeutics delivered *via* the circulation, and research supports that psychosocial stressors can affect cancer outcomes (80–82).

Antitumor host defense may provide an explanation, at least in part, for age- and/or stress-related differences, as it is well established that inflammation, innate and adaptive immunity regulates tumor progression (92–95). The numbers of tumor infiltrating F4/80^+^ macrophages and CD8^+^ T cells reported in this study are consistent with those reported by Nasu and colleagues (94, 95). F4/80^+^ macrophages were the major leukocyte infiltrate into the RM-9 tumor. The number of intra-tumoral macrophages was lower by about 30% in old versus young non-stressed mice, but increased by ~30% in old, stressed mice. No significant correlation between RM-9 tumor weight and tumor macrophage numbers was observed; however, increased intratumoral macrophage density was associated with poor prognosis (96).

Consistent with a role for adaptive immunity in regulating RM-9 tumor growth, altered tumor CD4^+^ T cell number and/or phenotype can affect the regulation of CD8^+^ T cell priming (97, 98). IL-2-mediated CD8^+^ T cell activation, clonal expansion, and maintenance of memory effector function was required for eliciting effective and specific immunity against tumors (98, 99). Still, the role of psychosocial stress and interaction with age and the cellular immune response against prostate cancer progression remains unclear. Like CD8^+^ T cells, CD4^+^FoxP3^+^ Treg cell infiltration was low in all treatment groups, but both age and stress effects were revealed, as well as relationships with circulating corticosterone and CD4^+^ T cell and macrophages tumor infiltration.

Few clinical studies have investigated links between psychosocial stressors on cytokine profiles in patients with PCa (100–102). Our data highlight the importance of using old animals to study PCa, as well as looking specifically at age-related differences in behaviors and physical response to the tumor. Human subjects participating in clinical studies are generally older men, since PCa is an age-related disease. Understanding age-related anxiety-like and depression-like behaviors and the subsequent effects of patient’s attitudes on cancer can lead to better therapeutic interventions for these older men. PCa studies using animal models tend to use young mice as subjects, which our data suggest may lead to invalid conclusions. Our data further supports that identifying age-related differences will enable researchers to discern valid parameters when using younger mice.

Future studies should include control animals to determine how the tumor affects behavior and possibly masks some of the response to other stressors. One of the limitations of this research is the lack of non-tumor groups with or without exposure to stress, which prevents assessment of surgery-related stress. Additionally, refined methods that increase the time interval between tumor inoculation and cancer-induced mortality are needed to optimize this model for studying how physiological reactions to stress vary with age and their consequences for behavior and tumor immunity.

To summarize, in the present study, all mice displayed increased anxiety-like symptoms above baseline measures. Age and stress affected general activity levels, and old mice demonstrated greater depression-like behaviors. Critically, age correlated with tumor size, as tumor growth was greater in old than young mice, although there were no age-related differences in tumor TGF-β1 or VEGF levels. Furthermore, although our model did not demonstrate that stress affected tumor size *per se*, the model was successful in showing that: (a) behavior was affected by stress; (b) stress hormone levels increased due to isolation and restraint-stressing, and (c) both age and stress differentially affected intratumoral leukocyte infiltration in a complex way possibly attributed to unique cytokine profiles. Differences in the infiltration of immune cell subsets in young and old mice strongly suggest age-dependent mechanisms in tumor immunity. Collectively, these findings highlight the importance of using age-relevant orthotopic models in understanding mind-body interactions on PCa outcome in the development of effective immune-based treatments in PCa that are prevalent in the elderly population.

## Data Availability Statement

The data that support the findings of this study are available from the corresponding author, DLB, upon reasonable request.

## Ethics Statement

The animal study was reviewed and approved by Loma Linda University Institutional Animal Care and Use Committee (IACUC).

## Author Contributions

DB: conceptualization, data curation, formal analysis, funding acquisition, investigation, methodology, project administration, resources, supervision, validation, visualization, writing original draft, review and editing. MD: data curation, formal analysis, investigation, methodology, manuscript writing reviewing and editing. CM: data curation, formal analysis, funding acquisition, investigation, investigation, methodology, validation, manuscript review and editing. PG: data curation, formal analysis, investigation, methodology, validation, manuscript review and editing. DL: formal analysis, investigation, manuscript review and editing. DG: conceptualization, investigation, methodologies, resources, manuscript review and editing. RH: conceptualization data curation, formal analysis, funding acquisition, investigation, methodology, project administration, resources, supervision, validation, visualization, manuscript writing original draft, review and editing. All authors contributed to the article and approved the submitted version.

## Funding

This research was supported by the National Cancer Institute at the National Institutes of Health [R21CA116698].

## Conflict of Interest

The authors declare that the research was conducted in the absence of any commercial or financial relationships that could be construed as a potential conflict of interest.

## Publisher’s Note

All claims expressed in this article are solely those of the authors and do not necessarily represent those of their affiliated organizations, or those of the publisher, the editors and the reviewers. Any product that may be evaluated in this article, or claim that may be made by its manufacturer, is not guaranteed or endorsed by the publisher.
